# Predictors for the uptake of optimal doses of sulfadoxine-pyrimethamine for intermittent preventive treatment of malaria during pregnancy in Tanzania: further analysis of the data of the 2015–2016 Tanzania demographic and health survey and malaria indicator survey

**DOI:** 10.1186/s12936-021-03616-2

**Published:** 2021-02-06

**Authors:** Vivian Mushi, Christopher H. Mbotwa, Abdallah Zacharia, Theresia Ambrose, Fabiola V. Moshi

**Affiliations:** 1 Department of Parasitology and Medical Entomology, School of Public Health and Social Sciences, Muhimbili University of Health and Allied Sciences, P. O. Box 65001, Dar es Salaam, Tanzania; 2 Mbeya College of Health and Allied Sciences, University of Dar Es Salaam, Mbeya, Tanzania; 3 Department of Epidemiology and Biostatistics, School of Public Health and Social Sciences, Muhimbili University of Health and Allied Sciences, Dar es Salaam, Tanzania; 4 Department of Nursing and Midwifery, College of Health Sciences, University of Dodoma, Dodoma, Tanzania

**Keywords:** Intermittent preventive treatment, Malaria, Pregnancy, Sulfadoxine-pyrimethamine and tanzania

## Abstract

**Background:**

In Tanzania, the uptake of optimal doses (≥ 3) of sulfadoxine-pyrimethamine for intermittent preventive treatment of malaria (IPTp-SP) during pregnancy has remained below the recommended target of 80%. Therefore, this study aimed to investigate the predictors for the uptake of optimal IPTp-SP among pregnant women in Tanzania.

**Methods:**

This study used data from the 2015–16 Tanzania demographic and health survey and malaria indicator survey (TDHS-MIS). The study had a total of 4111 women aged 15 to 49 who had live births 2 years preceding the survey. The outcome variable was uptake of three or more doses of IPTp-SP, and the independent variables were age, marital status, education level, place of residence, wealth index, occupation, geographic zone, parity, the timing of first antenatal care (ANC), number of ANC visits and type of the health facility for ANC visits. Predictors for the optimal uptake of IPTp-SP were assessed using univariate and multivariable logistic regression.

**Results:**

A total of 327 (8%) women had optimal uptake of IPTp-SP doses. Among the assessed predictors, the following were significantly associated with optimal uptake of IPTp-SP doses; education level [primary (AOR: 2.2, 95% CI 1.26–3.67); secondary or higher education (AOR: 2.1, 95% CI 1.08–4.22)], attended ANC at the first trimester (AOR: 2.4, 95% CI 1.20–4.96), attended ≥ 4 ANC visits (AOR: 1.9, 95% CI 1.34–2.83), attended government health facilities (AOR: 1.5, 95% CI 1.07–1.97) and geographic zone [Central (AOR: 5, 95% CI 2.08–11.95); Southern Highlands (AOR: 2.8, 95% CI 1.15–7.02); Southwest Highlands (AOR: 2.7, 95% CI 1.03–7.29); Lake (AOR: 3.5, 95% CI 1.51–8.14); Eastern (AOR: 1.5, 95% CI 1.88–11.07)].

**Conclusions:**

The uptake of optimal IPTp-SP doses is still low in Tanzania. The optimal uptake of IPTp-SP was associated with attending ANC in the first trimester, attending more than four ANC visits, attending government health facility for ANC, having primary, secondary, or higher education level, and geographic zone. Therefore, there is a need for health education and behavior change interventions with an emphasis on the optimal use of IPTp-SP doses.

## Background

Malaria in pregnancy is still a major public health problem causing maternal, fetus, and neonatal adverse health effects such as spontaneous abortion, maternal anaemia, stillbirth, premature birth, low birth weight, and maternal death [1]. Among the *Plasmodium s*pecies, *Plasmodium falciparum* is the leading cause of maternal illness, and low birth weight due to malaria, especially in Africa [2]. The estimates showed that 125 million pregnant women are at risk of acquiring malaria globally with 10,000 maternal deaths and 200,000 neonatal deaths annually as a result of malaria in pregnancy [3]. Also, malaria accounts for 18% of severe anaemia in pregnancy [4]. The occurrence of malaria in pregnancy has been associated with low gestational age, low maternal age, low parity, non-uptake of SP during pregnancy, level of knowledge on malaria prevention, place of residence of a woman, and household wealth status [5, 6].

The World Health Organization (WHO) recommends interventions such as the use of intermittent preventive treatment in pregnancy with sulfadoxine-pyrimethamine (IPTp-SP) and folic acid supplementation as a part of antenatal care services to prevent and treat malaria and anaemia during pregnancy in areas with moderate to high malaria transmission. However, the IPTp-SP should be combined with the use of insecticide-treated nets (ITNs) and effective case management of malaria [2]. The uptake of SP should starts as early as possible in the second trimester (13 weeks), and pregnant women should receive at least 3 doses with an interval of one month apart during the pregnancy [7]. The use of less than recommended doses of SP is less beneficial in the prevention of maternal and fetus/neonatal adverse health effects as it has been proven that the use of three or more doses to have beneficial effects against malaria-related health effect to both maternal and fetus/neonatal [1].

The evidence shows that the scale-up of malaria prevention interventions (IPTp and ITNs) in 25 malaria-endemic African countries have lowered neonatal mortality and low birth weight by 18% and 21%, respectively [3]. Tanzania is one of the malaria-endemic countries with approximately 1.7 million pregnant women at high risk of malaria infection [8]. The trend of malaria prevalence among pregnant women in Tanzania has been fluctuating with the highest of 10.3% in 2014 and the lowest of 6.8% in 2017 [9]. It was observed that despite the low prevalence of malaria in some areas, such as in Zanzibar Island, 0.8% of the pregnant women had malaria parasitaemia during delivery [10].

Tanzania opted for the IPTp-SP policy in 2001, which recommended the use of at least two doses of SP, and for the revised policy of using ≥ 3 doses of IPTp-SP in 2013. The Tanzania demographic and health survey and malaria indicator survey of 2015/16 has reported the uptake of sulfadoxine-pyrimethamine (SP) among pregnant women to be 68% for the first dose, 35% for the second dose and 8% for the third dose [11]. The uptake of optimal doses (at least three or more doses) of SP among pregnant women in Tanzania is still low compared to the required optimal coverage of at least 80% [3]. The low coverage of IPTp-SP was also observed in sub-Saharan Africa with the median coverage of 64%, 38%, and 23% for first, second and third doses, respectively [12].

Globally, studies have reported potential determinants associated with the uptake of at least two doses of SP to be; the age of a woman, place of residence, education level of a woman, household social-economic status, knowledge of a woman about malaria and IPTp-SP, number and timing of antenatal care (ANC) visit [12–17]. It was observed that educated and wealthier women were knowledgeable about IPTp and more likely to receive the SP [18]. Also, the uptake of SP was higher for women who attended 3 to 4 ANC visits and started ANC visits in their first or second trimester compared to those who started in the third trimester [13, 16, 17, 19].

There is a paucity of nationally representative studies in Tanzania that identified the predictors for the uptake of optimal doses (≥ 3) of IPTp-SP. Therefore, this study aimed to analyse the predictors for the uptake of the optimal doses of SP among pregnant women who gave birth two years preceding the 2015–16 TDHS-MIS. The findings shall inform the policymakers and programme implementers on the necessary changes to be done to improve the uptake of SP to optimal doses as recommended by the WHO.

## Methods

### Study design and data source

This study was a cross-sectional study utilizing the TDHS-MIS dataset of 2015–16. This was a sixth in a series of nationally representative household surveys conducted in Tanzania. It was conducted from August 2015 to February 2016 to provide up to date data on the areas of health, population (demographics), and nutrition. It was done under the collaboration of the Demographic and Health Surveys Program, National Bureau of Statistics, Office of Chief Government Statistician and Ministries of health in Tanzania Mainland and Zanzibar.

### Study population and sample size

The study population was women of reproductive age (15–49) who had a live birth in the two years before the survey. The data was extracted from women's individual recode file (TZIR7BFL), which had a total sample size of 4128 women who gave birth two years preceding the survey. However, 17 women were dropped because of incomplete information on the outcome variable. Therefore, the total sample size used for this study was 4111 women (unweighted sample) and 4056 women (weighted sample).

### Sampling technique

The 2015–16 TDHS-MIS employed stratified two-stage cluster sampling. The first stage involved the selection of clusters contained enumeration areas in which 608 clusters were selected. The second stage involved the systematic selection of the households from the 608 selected clusters, in which 22 households were selected in each cluster. This sampling technique produced a probability sample of 13,376 households whereby only 12,767 households were occupied. In the occupied households, a total number of 13,266 women were interviewed, and out of the interviewed women, 4128 gave birth 2 years before this survey. Hence, they were eligible for inclusion in this study [11].

### Measurement of variables

The variables associated with the uptake of IPTp were extracted from women’s data set based on the literature review. The outcome variable was uptake of three or more doses of IPTp-SP. The explanatory variables were socio-demographic characteristics (age, marital status, level of education, place of residence, the geographical zone of residence, wealth status) and obstetric characteristics (parity, the timing of first ANC visit, number of ANC visits, and type of health facility used for ANC services). The summary of variable definitions and categories are shown in Table 1.

**Table 1:** Variables extracted and used for this study

Variable	Definition	Categories
Outcome variable		
Uptake of IPTp-SP	Three or more doses of doses of IPTp-SP is optimal	< 3 doses
		≥ 3 doses (optimal)
Explanatory variables		
Age	Woman’s age group	15–19
		20–24
		25–29
		30–34
		35–39
		40–44
		45–49
Marital status	Woman’s marital union status	Never in union
		In union
		No longer in union
Education level	Woman’s highest level of education	No formal education
		Primary education
		Secondary education and beyond
Place of residence	Area or place where a woman was residing	Urban
		Rural
Occupation	Woman’s occupation	Unemployed
		Self-employed
		Employed
Wealth index status	Household’s wealth index from which a woman is coming	Poorest
		Poor
		Middle
		Rich
		Richest
Parity	Number of live births that a woman had ever had	1
		2
		3 +
Timing of 1st ANC visit	Gestational age (pregnancy) age in months at which a woman visited ANC for first time	First trimester
		Second trimester
		Third trimester
Number of ANC visits	Number of ANC visits a pregnant women made during her gestation period	01-Mar
		4 +
Type of facility for ANC visit	Type of ownership of a facility (Government or non-government) that a woman was attending for ANC	Non-government
		Government
Geographical zone	Geographical zone from which a woman was residing	Western
		Northern
		Central
		Southern highlands
		Southern
		South west highlands
		Lake
		Eastern
		Zanzibar

### Data management and analysis

The data were extracted, cleaned, and analysed using STATA version 14 (STATA Corp, College Station, TX, USA). Descriptive statistical analysis was conducted first on socio-demographics and obstetric characteristics to obtain frequencies and proportions. Then, univariate logistic regression was conducted to identify the variables for the multivariable logistic regression model. All explanatory variables with a p-value < 0.25 on univariate analysis were subjected to multivariable logistic regression for further analysis of the association to obtain adjusted odds ratios. To account for the differences in sampling probabilities across the clusters and strata, sample weighting was used to adjust for the cluster sampling design.

### Ethical consideration

This study used secondary data without involving any human subjects. Therefore, no formal ethical approval was required. However, the Tanzania Demographic and Health survey was conducted after approval from national and international review boards including; the National Institute of Medical Research, Zanzibar Medical Research Ethical Committee, Institutional Review Board of Inner City Fund, and the Centers for Disease Control and Prevention in Atlanta. All women interviewed were requested to provide verbal informed consent before the commencement of the study. The permission to use the IPTp-SP data was sought and obtained from the DHS program.

## Results

### Socio-demographic characteristics of the study respondents

This study included 4111 women, more than a quarter (27.3%) were between the ages of 20–24 years. The majority of the respondents (75.9%) lived in rural areas, and 83% were in a union. More than half of the respondents (59.6%) had attained primary education, and 51.4% were self-employed (Table 2).

**Table 2:** Socio- demographic characteristics of the study respondents (n = 4111)

Socio-demographic variable	Frequency (n)	Percentage (%)
Age (years)		
15–19	480	11.7
20–24	1124	27.3
25–29	978	23.8
30–34	715	17.4
35–39	518	12.6
40–44	245	6
45–49	51	1.2
Marital status		
Never in union	311	7.6
In union	3413	83
No longer in union	387	9.4
Education level		
No formal education	795	19.3
Primary	2450	59.6
Secondary +	866	21.1
Place of residence		
Urban	991	24.1
Rural	3120	75.9
Wealth index status		
Poorest	927	22.5
Poorer	828	20.1
Middle	780	19
Richer	888	21.6
Richest	688	16.7
Occupation		
Unemployed	818	19.9
Self-employed	2113	51.4
Employed	1180	28.7
Geographic zone		
Western	408	10
Northern	306	7.4
Central	420	10.2
Southern highlands	280	6.8
Southern	151	3.7
South west highlands	457	11.1
Lake	1128	27.4
Eastern	373	9.1
Zanzibar	588	14.3

### Predictors for the uptake of optimal doses of IPTp-SP during pregnancy

The overall uptake of optimal doses of IPTp-SP was 8%. The uptake of ≥ 3 doses of SP increased by level education ranging from 3.9% for women with no formal education to 10.7% for women with secondary and above education. The women who started ANC in the first trimester (12.6%), and attended four or more ANC visits (11.7%), had higher uptake of optimal doses compared to women who started ANC late and attended 1–3 ANC visits. Also, the uptake of optimal doses of SP was high for women who attended government health facilities (10.4%) as compared to those who attended non-government facilities (7.4%) during their ANC visits (Table 3).

**Table 3:** Predictors of the Optimal Uptake of IPTp-SP (≥ 3 doses) during pregnancy

Variables	N (weighted)	Took 3 + doses	^a^COR (95% CI)	p-value	^b^AOR (95% CI)	p-value
		N (%)				
Overall	4056	327 (8.1)				
Age (years)						
15–19 (ref)	531	35 (6.5)	1.0			
20–24	1114	102 (9.1)	1.3 (0.91–2.26)	0.283		
25–29	963	67 (6.9)	1.1 (0.65–1.74)	0.812		
30–34	696	58 (8.3)	1.3 (0.77–2.17)	0.325		
35–39	487	46 (9.5)	1.5 (0.88–2.55)	0.260		
40–44	231	16 (7.2)	1.1 (0.55–2.20)	0.780		
45–49	34	3 (10.5)	1.7 (0.52–5.35)	0.388		
Marital status						
Never in union (ref)	351	40 (11.4)	1.0			
In union	3307	256 (7.7)	0.6 (0.53–1.17)	0.339		
No longer in union	398	31 (7.7)	0.7 (0.37–1.21)	0.436		
Education level						
No formal (ref)	762	30 (3.9)	1.0		1.0	
Primary	2,607	224 (8.6)	2.3 (1.42–3.70)	0.001	2.2 (1.26–3.67)	0.005
Secondary +	687	73 (10.7)	2.9 (1.71–5.00)	< 0.001	2.1 (1.08–4.22)	0.029
Residence						
Rural (Ref)	1128	131 (11.6)	1.0		1.0	
Urban	2928	196 (6.7)	1.8 (1.33–2.53)	< 0.001	1.1 (0.66–1.90)	0.677
Occupation						
Unemployed (ref)	774	63 (8.1)	1.0			
Self-employed	2160	150 (7.0)	0.8 (0.58–1.23)	0.383		
Employed	1122	114 (10.1)	1.3 (0.88–1.84)	0.197		
Wealth index						
Poorest (ref)	966	58 (6.0)	1.0		1.0	
Poorer	857	66 (7.7)	1.3 (0.84–2.05)	0.227	1.2 (0.78–1.94)	0.364
Middle	771	48 (6.2)	1.0 (0.69–1.58)	0.851	0.8 (0.54–1.25)	0.352
Richer	779	66 (8.4)	1.4 (0.92–2.27)	0.110	1.0 (0.62–1.75)	0.886
Richest	683	89 (13.1)	2.4 (1.52–3.66)	< 0.001	1.3 (0.63–2.52)	0.518
Parity						
1	1110	103 (9.3)	1.4 (1.00–1.96)	0.051	1.2 (0.80–1.71)	0.412
2	782	77 (9.8)	1.5(1.08–2.09)	0.017	1.3 (0.92–1.82)	0.143
3 + (ref)	2164	147 (6.8)	1.0		1.0	
Timing of 1st ANC visit						
1st trimester	928	116 (12.6)	4.7 (2.55–8.81)	< 0.001	2.4 (1.20–4.96)	0.014
2nd trimester	2691	198 (7.3)	2.6 (1.41–4.85)	0.002	1.8 (0.93–3.47)	0.080
3rd trimester (ref)	434	13 (2.9)	1.0		1.0	
Number of ANC visits						
1–3 (ref)	2054	91 (4.4)	1.0		1.0	
4 +	1994	233 (11.7)	2.9 (2.12–3.86)	< 0.001	1.9 (1.34–2.83)	< 0.001
Health facility for ANC						
Non-government (ref)	3199	238 (7.4)	1.0		1.0	
Government	857	89 (10.4)	1.5 (1.09–1.94)	0.012	1.5 (1.07–1.97)	0.017
Geographic zone						
Western (ref)	525	11 (2.1)	1.0		1.0	
Northern	384	12 (3.0)	1.5 (0.53–4.19)	0.443	0.9 (0.32–2.79)	0.911
Central	477	51 (10.8)	5.7 (2.40–13.7)	< 0.001	5.0 (2.08–11.9)	< 0.001
Southern highlands	215	17 (8.1)	4.2 (1.76–10.2)	0.001	2.8 (1.15–7.02)	0.023
Southern	148	16 (10.6)	5.7 (2.19–14.7)	< 0.001	3.8 (1.41–10.1)	0.008
South west highlands	400	27 (6.7)	3.4 (1.30–8.87)	0.013	2.7 (1.03–7.29)	0.043
Lake	1237	101 (8.2)	4.2 (1.84–9.73)	0.001	3.5 (1.51–8.14)	0.004
Eastern	566	86 (15.3)	8.6 (3.73–19.7)	< 0.001	4.6 (1.88–11.1)	0.001
Zanzibar	104	6 (5.7)	2.9 (1.25–6.71)	0.014	2.1 (0.80–5.36)	0.133

^*a*^*COR* Stands for Crude Odds Ratios, ^*b*^*AOR* Stands for Adjusted Odds Ratios

The results of univariate logistic regression analysis (crude odds ratios) show that woman’s education level, place of residence, wealth index status, parity, the timing of first ANC visit, number of ANC visits, type of ANC facility visited, and geographical zones were significant associated with the of optimal uptake of IPTp-SP. After adjusting for the confounders, the predictors independently associated with optimal uptake of IPTp-SP were; education level, the timing of the first ANC visits, number of ANC visits, type of health facility for ANC visits, and geographical zone (Table 3).

The women who started ANC visits during the first and second trimesters had high odds (4.7 and 2.6) respectively for optimal uptake of SP, compared to women who started ANC visits during the third trimester. Also, the women who had four or more visits were 2.9 more likely to take optimal doses of SP, compared to women who had less than four visits. Furthermore, the women who attended government health facilities were 1.5 times more likely to take optimal doses of SP compared to women who attended non-government facilities for ANC visits (Table 3).

## Discussion

This study used data from TDHS-MIS 2015/16 to analyse the predictors for the uptake of optimal doses of SP (three or more doses) among pregnant women. The uptake of three or more doses of SP was reported to be 8% countrywide, which is still low than the recommended coverage of 80% from WHO and Roll Back Malaria (RBM) benchmark target [3]. The observed low uptake of the optimal doses of SP in TDHS-MIS 2015/16 could be contributed partially by updates of the IPTp-SP policy in 2013, from at least two doses to ≥ 3 doses of SP. Several studies conducted in other sub-Saharan countries have reported the low uptake of the optimal doses of IPTp-SP [20–23]. Hence, the urgent need to plan effective strategies to improve IPTp-SP coverage and uptake in sub-Saharan Africa.

The predicators for the uptake of optimal doses of SP were; geographical zones, the education level (primary, secondary or higher education), attending ANC in the first trimester of pregnancy, attending ANC visit more than four times, and attending government health facility for ANC services. Pregnant women who attained at least primary education were likely to receive optimal doses of SP, compared to those with informal education. This is because educated pregnant women could be aware and knowledgeable on the importance and benefits of using SP for malaria prevention during pregnancy. Similarly, the findings from Nigeria [24], Malawi [21, 25], Ghana [22], and Zimbabwe [23] showed that the knowledge on the SP and on the consequences of not taking IPTp-SP as a facilitator toward the uptake hence the association between education level and the likelihood of the uptake of three or more doses of SP for malaria prevention during pregnancy.

Pregnant women who registered and attended ANC clinics in their first trimester received optimal doses of SP compared to women attended ANC clinic in the third trimester. A possible explanation could be; attending ANC clinics in the first trimester gives the room for pregnant women to attend ANC more than four times hence higher chances for start taking SP doses in their second trimester as required. Also, it has been predicted in several studies conducted in Zimbabwe, Sierra Leone, Malawi, Nigeria, and Uganda that early booking and attending of first ANC in the first or second trimester has an association with receiving optimal doses of SP while late attending to ANC clinic results in lower uptake of SP doses [6, 15, 23, 24, 26].

A significant relationship between the number of ANC visits and uptake of optimal doses of SP was observed in our study. The pregnant women who attended at least four ANC visit received optimal doses of SP compared to women with few attendances. The more the pregnant women attend the clinic, the higher the exposure toward health information on IPTp-SP hence the higher likelihood of receiving optimal doses of SP. The findings are consistent with the studies conducted in Malawi, Ghana, and Cameroon [21, 27, 28]. Also, attending ANC visits only once or at late, such as after 36 weeks where pregnant women cannot receive three or more doses of SP, were observed as a barrier towards the uptake of optimal doses of SP. Therefore, the urge to raise awareness among pregnant women on the importance of early and adequate attendance to ANC clinics to receive optimal doses of SP for malaria prevention is important.

Attending government (public) health facilities for ANC was found to influence the uptake of optimal SP doses among pregnant women compared to those who attended private clinics. The government of Tanzania, through the Ministry of Health, usually provides regular in-service training to both public and private ANC clinics on the provision of IPTp-SP to pregnant women. The clinics are responsible for sending the staff for the training. However, private clinics were observed to send a few numbers of staff compared to public clinics for training [29]. The low proportion of trained staff at the private clinics could affect the provision of SP and hence the uptake of optimal doses of SP among pregnant women. The high uptake of optimal doses of SP at the public clinics could be a result of sensitization of the SP uptake under direct observation therapy (DOT) and seriousness of following SP administration protocol. It was noticed that in some private clinics, pregnant women were allowed to take the drugs at home. Hence, compromise the optimal uptake of SP doses. The findings are in accordance with a study conducted in Ghana, which found poor adherence to DOT in private health facilities as one of the obstacles towards the uptake of optimal doses of SP [30].

Geographical zones were also the predictors for the optimal uptake of SP doses. Being a resident of regions that belong to Central, Eastern, Southern, Lake, Southern highlands, and South West highlands was significantly associated with the optimal uptake of SP doses compared to the residents of Zanzibar and Northern zones. This might be contributed by the level of malaria endemicity in different zones. In the zones with a high or moderate level of malaria transmission, possibly the awareness and emphasis on SP uptake could be higher due to the higher risk of contracting malaria that’s why pregnant women in those zones had higher odds of taking optimal doses of SP compared to those residing at Zanzibar and Northern zones where there is a low level of malaria transmission. The observed findings are consistent with another study conducted in Tanzania, which showed that pregnant women residing in Eastern and Coastal regions had higher odds of optimal uptake of SP [26].

This study had the following limitations; the data analysis was limited only to the variables captured on demographic and health survey questionnaire, some of the important variables that could influence the uptake of optimal doses of IPTp-SP were not captured, for example, socio-cultural factors, knowledge of health care providers and availability of SP in ANC clinics hence hindered full exploration of other important variables. Response (recall) bias was another limitation; the data collection was based on self-reported experiences of the past two years. Hence, due to response bias, there was a possibility of over-or- underestimation of the responses.

## Conclusions

The uptake of optimal doses of SP among pregnant women in Tanzania is still below the WHO recommendations. The identified predictors for optimal uptake of SP were primary and secondary or higher education level, attending ANC in the first trimester, attending ≥ 4 ANC visits, attending a government health facility for ANC services, and being a resident of any geographical zone except for Northern and Zanzibar zones. The alarming findings indicate the urgent need to improve the uptake of optimal doses of SP among pregnant women in Tanzania. Therefore, the need for health education, social, and behavior change interventions with an emphasis on the earlier attendance to ANC clinics and on the optimal use of IPTp-SP doses. The mentioned interventions will help to improve the awareness and knowledge of the optimal use of IPTp-SP among pregnant women in Tanzania.

## Data Availability

All the data used are available upon request from demographic and health surveys website. The questionnaire used for analysis was women questionnaire which was appended in TDHS-MIS report of 2015/16.

## Abbreviations

ANC: Antenatal care
DOT: Direct observation therapy
IPTp-SP: Intermittent preventive treatment in pregnancy with sulfadoxine-pyrimethamine
ITNs: Insecticide-treated nets
RBM: Roll back malaria
SP: Sulfadoxine-pyrimethamine
TDHS-MIS: Tanzania demographic and health survey and malaria indicator survey
USAID: United States Agency for International Development
WHO: World Health Organization
